# Determination of Neonicotinoids in Honey Samples Originated from Poland and Other World Countries

**DOI:** 10.3390/molecules25245817

**Published:** 2020-12-09

**Authors:** Magdalena Ligor, Małgorzata Bukowska, Ileana-Andreea Ratiu, Renata Gadzała-Kopciuch, Bogusław Buszewski

**Affiliations:** 1 Department of Environmental Chemistry and Bioanalytics, Faculty of Chemistry, Nicolaus Copernicus University, 7 Gagarina Str., 87-100 Torun, Poland; malgorzatabukowska72@gmail.com (M.B.); rgadz@umk.pl (R.G.-K.); 2 Interdisciplinary Centre of Modern Technologies, Nicolaus Copernicus University, 4 Wileńska Str., 87-100 Torun, Poland; andreea_ratiu84@yahoo.com; 3 “RalucaRipan” Institute for Research in Chemistry, Babes-Bolyai University, 30 Fantanele, RO-400239 Cluj-Napoca, Romania

**Keywords:** neonicotinoid residues, honey, QuEChERS, UHPLC/UV

## Abstract

A method development for determination of neonicotinoid residues in honey samples was developed. The proposed methodology consisted in QuEChERS (Quick, Easy, Cheap, Effective, Rugged and Safe). That was used for sample preparation and UHPLC/UV (ultra-performance liquid chromatography with ultraviolet detection) utilized for chromatographic analysis. The developed method proved to be sensitive, with LOD (Limit of detection) value in the range of 60.80 to 80.98 ng/g hence LOQ (Limit of quantification) value was in the range of 184.26 to 245.40 ng/g. The method has tested on Polish honey and applied to honey from various countries (Bulgaria, Czech Republic, France, Greece, Italy, Portugal, Romania, Australia, Brazil, Cameroon, Russia, USA and Turkey). Several honey types were tested, while physicochemical properties of all honeys and were investigated. The methodology for general characterization of pollen grains originated from selected plants, to confirm the type of honey was also presented. There was a total lack of the mentioned neonicotinoids in sunflower honey. Except of this, only two samples of rapeseed and two samples of acacia honey (from Poland and Romania) were neonicotinoids free. In 19 samples the targeted pesticides were detected above LOQ. In all other investigated samples, the neonicotinoids were found at least at the LOD or LOQ level.

## 1. Introduction

Honey is an important natural sweetener produced by bees and obtained from floral nectar. The market for honey is anticipated to increase in the near future due to the consumers’ awareness of the beneficial properties of this foodstuff. However, even if the world exports of honey increased, bees’ colony numbers have remained stable and productivity per hive of authentic pure honey has declined. The reliable approaches to detect honey adulteration, concern the production process, with main issues related to sugar syrup addition, filtration, thermal treatment and water content as well as and the geographical and/or botanical origin of honey it are very important issues nowadays [1].

Among the major components of honey, the next can be reported: sugars, water, nitrogenous substances and elements, proteins, organic acids, polyphenols [2,3,4]. The variable carbohydrates content in honey was observed and it is dependent mostly on the floral source of the honey [5,6]. Moreover, the sugar profiles’ including fructose, glucose, sucrose, maltose contents and glucose and water ratios can be useful for the characterization of unifloral honeys. The complex mixture of oligosaccharides in honey may be useful in determining its floral origin [7]. Usually honey is characterized by low pH which is related to the presence of variety of organic acids (e.g., acetic, butyric, citric, formic, gluconic, lactic, malic, pyroglutamic and succinic) [8]. Antibacterial properties of honey against various pathogens have been reported as well [9]. Nevertheless, the trace amount of artificial substances in honey and pollen is a real problem [10]. Many industrial practices of agribusinesses, like synthetic fertilizers, pesticides and herbicides have harmed organisms in the soil and pollinators [11,12,13,14,15]. Some farmers hold the view that the decision to withdraw the mortar could be unnecessary. Their lack caused the necessity of applying sprays, which, in the opinion of agricultural organizations, more than mortars increase the threat to bees and burden the environment. In addition, they affect the increase in production costs and contribute to the increase of pests [16]. The pesticides and their residues in hive products, leaded in colony disease and collapse [17,18]. The additive and synergistic effects of multiple pesticide exposures that contributed to declining honey bee health are well documented [12,19,20,21].

Among compounds labeled as toxic to pollinators an important group are neonicotinoids. Five well known neonicotinoids are: imidacloprid (moderately toxic and highly toxic to bees and other beneficial insects), acetamiprid (low acute toxic, it has shown population-level effects on honeybees), clothianidin (neurotoxic and highly toxic to bees and other non-target insects), thiacloprid (low doses are highly toxic to honeybees, causes physiological problems in fish within water environments), thiamethoxam (systemic insecticide, absorbed and transported to all parts of the plant and while considered moderately toxic, harmful to bees, aquatic and soil organisms) [22,23,24]. 

From the analytical point of view, for determination of neonicotinoids in honey samples one of the most appropriate technique is high performance liquid chromatography (HPLC) in various counterparts [25,26,27,28,29,30]. The sample preparation step and selection of a suitable extraction method are equally important part of methodology applied for the separation of neonicotinoid residues in honey. Liquid—liquid and/or liquid—solid extraction methods including solid phase extraction (SPE) and other methods—QuEChERS (solid phase extraction) have been described [31,32,33]. Some methods require the use of modern equipment and various laborious methods for isolating and determining the analytes. All these may influence the required time of analysis and performances. The detection system is also an important part of a developed methodology. Consequently, many types of detectors are proposed, such as: UV/Vis, diode array detector (DAD), light scattering, corona discharge, fluorescence, conductivity, electrochemical, radioactivity, chemiluminescent as well as the tandem mass spectrometry detection techniques (MS/MS) [27,28,30].

Among insecticides that have been found in honey and widely used in agriculture, occur substances belonging to the neonicotinoid group [24,34,35,36]. It was confirmed that the honey contaminated with neonicotinoids is not usually harmful to people, however, their presence is threatening the bee safety [24,37,38,39,40]. Numerous studies indicate that neonicotinoids act on the nicotinic acetylcholine receptors (nAChRs) in the central nervous system, which leads to elimination of insects. There is evidence indicating that neonicotinoids may adversely affect honey bees and other pollinators, via impairments on learning and memory and in the consequence desist an ultimately foraging [37,39]. Nevertheless, the specific mechanisms linking activation of the nAChR to adverse effects on learning and memory are not fully confirmed. In addition, it still lacks clear connections between observed impacts on individual bees and colony level effects [37,38,41,42]. Furthermore, insecticides contained in plants pollinated by bees are very dangerous for them—they cause problems with the bees and impair their ability to accumulate food [25,26]. That is why the research conducted all over the world is so important, allowing for getting as much data as possible, with particular emphasis on bees and other important pollinators [20,24,43].

In the current study a new method development for the qualitative and quantitative analysis of various honey samples has been presented. The developed method required to apply a QuEChERS method as a highly recoverable sample preparation method and UHPLC/UV for the final analysis. The proposed methodology could be successfully used for the separation and determination of persistent natural products impurities resulting from the exposure of bees to the environmental pollution. The obtained results allowed discussing the impact of the origin and geographical traceability for the concentration level of selected neonicotinoids in honey.

## 2. Results and Discussion

### 2.1. Differentiation of Honey’s Variety and the Presence of Flower Pollen Grains

In addition to its main components (mostly sugars), each type of honey also contains solids, including i.a. pollen of different plants, spores of fungi or algae and so on. The species of flower pollen found in a specific batch of honey can be used to determine the floral sources from which bees collected the nectar as well as the geographical origin of honey. Various classes and subclasses of honey contain different amounts of pollen, depending on factors such as:

- the type of floral source (in turn dependent on flower structure, pollen content in nectar and the way in which nectar is collected and processed by bees);
- time of day when bees collect pollen and mix it with nectar from proventriculus (dependent on hive location and distance to the floral source);
- the way how honey is extracted from honeycombs by beekeepers (the extracted honey can get mixed with bee pollen or residue of honey from other floral sources).

For bees and other pollinator insects, flower pollen is an important source of protein, fat, minerals and vitamins and thus it is necessary for their correct development. Flower pollen contains 20–40% proteins, 15–48% carbohydrates, 2–14% lipids, 1–5% minerals, approx. 1% vitamins, as well as enzymes, bactericidal and fungicidal substances, essential oils, hormones, organic acids and so forth [44]. 

Flower pollen is turned into a bee product known as bee pollen or bee bread. Foraging bees collect pollen from flowers and mix it with the secretions of their salivary glands or with nectar, filling pollen baskets on their hind legs and then transport the load to the hive. The workers in the hive store the collected pollen in honeycomb cells, wetting it with saliva and honey, turning it into small granules and packing them layer after layer. A filled cell is covered with honey and wax. Thus, prepared content of a cell undergoes lactic acid fermentation in anaerobic conditions, turning into bee pollen. This natural product is basic food for bees [44]. 

Most pollen grains present in honey come from entomophilic plants; however, bee products often also contain pollen from anemophilic plants, which can be responsible for certain allergy symptoms [45]. The presence of anemophilic plant pollen is affecting people allergic to airborne pollen present in the summer (when plants pollinate). Consequently, they may experience an allergic reaction after consuming honey. Allergy to honey is rare, usually observed in persons allergic to specific foods, plant pollen and bee venom.

In most cases it is caused by flower pollen proteins; allergy to proteins in bee secretions is much less frequent. Allergies to honey are often caused by pollen of the members of the *Compositae* family, or—less often—by pollen of grasses and trees [46].

In our study, pollen obtained from flowers of buckwheat, goldenrod, blue tansy and rapeseed was characterized with scanning electron microscopy (SEM). The samples were also assessed with Energy Dispersive X-ray Spectroscopy (EDX) to determine the contents of selected elements. Sample SEM images and EDX results are presented in Table 1.

The main factor on which the quality of honey depends is the type of flower source used by bees. Plant species growing in a given area influence the organoleptic characteristics of honey (taste, smell and color), its chemical composition and the specific properties of different monofloral honeys. The state of natural environment as well as agricultural techniques (use of fertilizers and plant protection products) are the environmental conditions that determine the quality of material collected and processed by bees. In turn, the quality of air and water has a significant impact on the life of a swarm (the environment inside the hive). Other factors influencing the quality of honey are related to extracting honey and preparing it for commercial sale, including heating (recrystallization), fermentation, filtration, sanitary conditions during extraction and so forth.

### 2.2. Method Development

The first step of proposed method consisted in the application of QuEChERS as a sample preparation method of honey samples for analysis by mean of UHPLC. Only in a few cases it was not necessary to carry out the purification step to obtain pure extracts of honey. As is commonly known, honey extract contains high concentration of carbohydrates, especially glucose and fructose, which can induce undesirable matrix effect. The purification step was necessary in most cases for honey extract preparation since the signals from impurities were still present after the extraction procedure was finished. These impurities made difficult the interpretation of results. That is why a mixture of the salts MgSO_4_ anhydrous and NaCl was added to the aqueous solution of honey. Moreover, mixtures of MgSO_4_ anhydrous, PSA and C18 have been applied for QuEChERS. Both PSA are useful in the removal of sugars, fatty acids, organic acids, lipids and some pigments. Moreover, in combination with Cl8, additional lipids and sterols could be removed. Also, C18 allows removing of long chain fatty compounds, sterols and other non-polar interferences, as previously demonstrated [47,48,49]. 

Regarding the chromatographic methodology necessary for qualitative and quantitative analysis a preliminary optimization was realized using a standard solution of imidacloprid (concentration level 1 µg/mL, solvent ACN/H_2_O 50/50). Five different columns (Acquity UPLC BEH C18, Sunshell PFP, Sunshell Phenyl, Discovery C18 and Discovery C8) were tested. The obtained results highlighted that Sunshell Phenyl column performed better than the other used. Generally, SunShell type of columns are core shell columns with inert surface due to inactivated silanol groups and high stability through special end-capping. The selected SunShell Phenyl column is characterized by appropriate pH stability (pH 2.5–10), higher loading capacity, low back pressure (in this case 85 bar), well supporting the applied temperature (45 °C) and with good performance (for imidacloprid approx. 9000 theoretical plates/meters).

**Figure 1:**
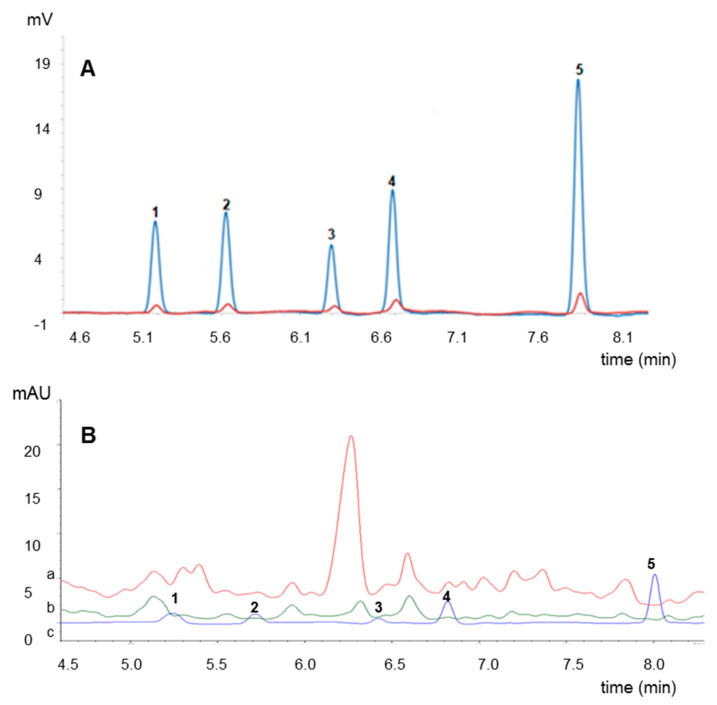
An exemplary chromatogram of standards solution (**A**) and chromatograms of neonicotinoids-free acacia honey samples (**B**) after: extraction process (a), extraction and purification (b), in comparison with a chromatogram of target compounds (c), all of them obtained at the wave length 245 nm; where 1—thiamethoxam, 2—clothianidin, 3—imidacloprid, 4—acetamiprid and 5—thiacloprid.

After the cleaning steps and the removal of interfering substances, the signals were separated from the baseline and the symmetry of the peaks was improved, which made easier the quantitative analysis carrying out. The obtained results of determination of neonicotinoids content in the examined honey have been compared to assess the quality of honey. An exemplary chromatogram of neonicotinoid standards (i.e., (1) thiamethoxam, (2) clothianidin, (3) imidacloprid, (4) acetamiprid and (5) thiacloprid) is presented in Figure 1A at minimum concentration level (1 = 99.69 ng/mL, 2 = 100.30 ng/mL, 3 = 99.50 ng/mL, 4 = 99.90 ng/mL, 5 = 149.85 ng/mL)—smallest peaks and maximum concentration (1 = 697.83 ng/mL, 2 = 702.10 ng/mL, 3 = 696.50 ng/mL, 4 = 699.30 ng/mL, 5 = 1748.25 ng/mL)—highest peaks. 

The developed method has been validated and it proved to be correct. Consequently, it allowed obtaining extracts form honey fully purified and free of inadvisable artifacts or unknown interfering substances Figure 1B.

Moreover, the recovery rate (RR) of the applied method has been calculated to each target compounds using the equation presented below Equation (1).

RR = C_1_/C_2_ × 100% (1)

where: C_1_ is the concentration of each neonicotinoid in extracts of honey after sample after applying the sample preparation procedure, C_2_ is the concentration of each compound in honey after the enrichment of honey sample by 1 mL or 0.6 mL of a standard mixture of acetonitrile (at concentration level 2.0 µg/mL for acetamiprid, imidacloprid, clothianidin, thiamethoxam and 5.0 µg/mL for thiacloprid).

**Figure 2:**
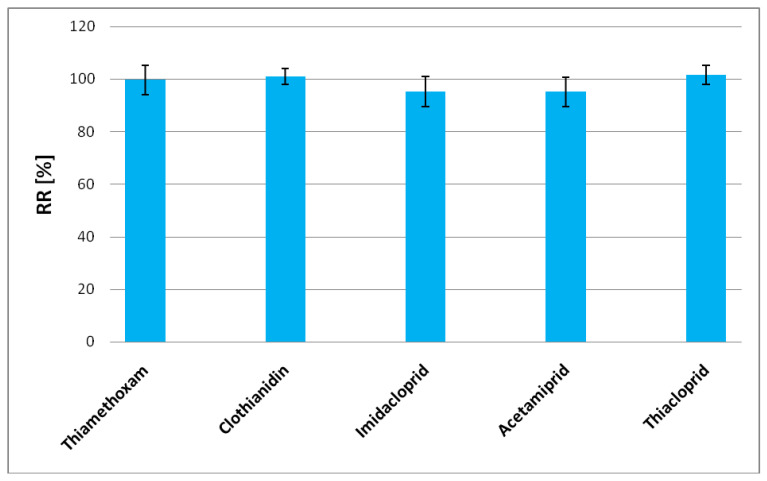
The recovery rates for thiamethoxam, clothianidin, imidacloprid, acetamiprid and thiacloprid (*n* = 6).

The recovery rate has been presented for each neonicotinoid in Figure 2. That proposed method used for the separation and determination of neonicotinoids from honey including the extraction part by use of QuEChERS and the analysis part by using UHPLC/UV. The method proved to be appropriate and gives in a short time reproducible results, confirming high extraction efficiency. Moreover, the linearity of each neonicotinoids was determined as the ratio of area versus concentration. Each level was repeated six times, during three different days. The regression coefficients were found R2 > 0.999 for all analytes. The results are presented in Table 2.

Honey samples fortificated all neonicotinoids for two levels: 1st level (120 ng/mL for four neonicotinoids and 300 ng/mL for thiacloprid) and 2nd level (200 ng/mL for four neonicotinoids and 500 ng/mL for thiacloprid). The precision of the method was below 10% for all analytes at both fortification levels. 

The sensitivity of the analytical method is defined as the lowest analyte concentration that can be measured with acceptable accuracy and precision. The adequate evaluation was performed as LOD, defined as the lowest amount of analyte in the sample, which can be detected but not quantified. The LOQ is defined as the lowest concentration which is sensitive to be determined quantitatively. LOD is equal to 3.3 times the quotient standard deviation of intercept and slope. LOQ is equal to10 times the quotient standard deviation of intercept and slope. Details of all these results are also reported in Table 2.

### 2.3. Correlation between Honey Types According to Their Physicochemical Properties

**Figure 3:**
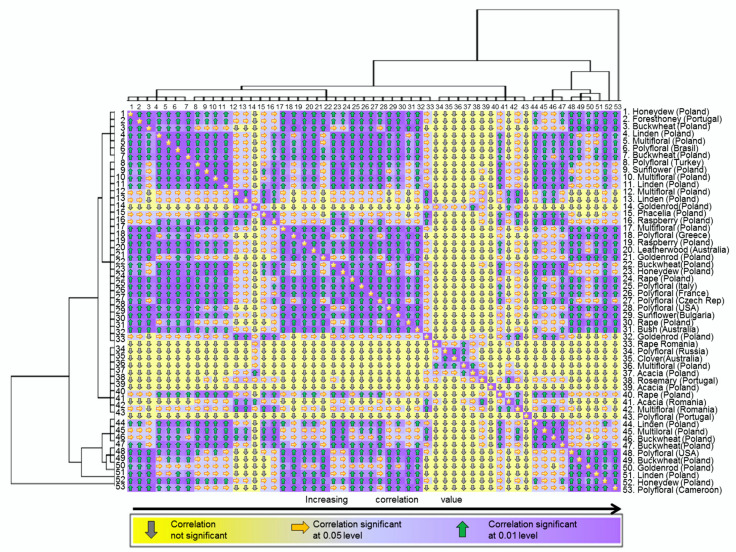
Heat map presenting the correlation between the 53 investigated honey samples.

Four physicochemical properties of analyzed honey were investigated, that is, pH, acidity, electrical conductivity and Pfund value. A Pearson moment correlation was conducted to check the correlation between samples and the level of significance. The correlation matrix was designed based on the hierarchical clusters’ analyses obtained using the method “average linkage between groups” within the interval “squared Euclidian distance” and is presented in Figure 3. The hierarchical clusters analyses lead to the formation of five clusters with the same level of significance, redistributed in turn to other six with different levels of significance. In the heat map presented in Figure 3 the correlation matrix is highlighted according with the pattern of clustering. Regarding the correlation values, we found just strongly positively correlation between the investigated samples (r(52) = 0.999, *p* = 0.01 up to r(52) = 0.950, *p* = 0.05) or no correlation. However, the clusters formed did not bring together similar honey types but honey with similar physicochemical properties. Moreover, it was previously demonstrated before that the physicochemical properties of the honey may vary depending on the season, area of plant cultivation and year rather than depending on the type of honey [50].

Honey pH is linked with the presence and growth of microorganisms; consequently, a low pH is associated with the absence of spoilage [50]. The recorded pH ranged between 3.31 and 4.27. No clusters formation according with the pH values could be identified. 

Pfund value established based on Pfund scale is a measurement of the honey color. In the 53 investigated samples the Pfund values ranged between 18 and 837 mm. The honey with light color, associated with the smallest Pfund values (samples 34 to 43) were fused together in a single cluster delimitating the heat map in two parts: the right side, including standard honeys and the left part which includes dark color honey (with Pfund values higher than 200 mm). Moreover, the honeys with light colors emphasized less correlation with others.

Honey acidity relates to the amount of organic acids, lactones, esters and ions of phosphates, sulphates and chlorides [50]. The maximum allowed value for acidity in honey is 50 meq/kg, while highest values were associated with the starting of fermentation process which leads to the production of alcohols, previously transformed in organic acids [50,51]. Seven of the 53 investigated samples (samples: 33, 44, 45, 46, 47, 52 and 53) exceeded the maximum allowed value for acidity. The sample 33 is distinguished in the heat map by making the transition between two main clusters. The samples 44 to 47 are all fused together in a single cluster in the left part of the map, while the samples 52 and 53 are closing the heat map in the left part, without joining any cluster. All these findings are indicating that the mentioned seven samples are presenting non-standard characteristics compared with others.

The electrical conductivity is an important compliance parameter for honey quality, the maximum allowed value being of 0.8 mS/cm [50,51]. In case of our samples, the electrical conductivity was between 0.03 up to 1.196 mS/cm. Four of the investigated samples exceeded the maximum allowed value; however, they did not cluster together.

### 2.4. The Neonicotinoids Content in Honey

The quantitative results obtained for all 53 samples of honey are presented in Table 3. Each sample of honey extract was analyzed in triplicate. The analysis was performed as follows: a complete set, including 53 honey samples (one sample of each type) was analyzed, then a second, followed by the third set were also prepared and analyzed. This was realized to obtain representative results and to ensure the inter-day repeatability of the method. 

**Figure 4:**
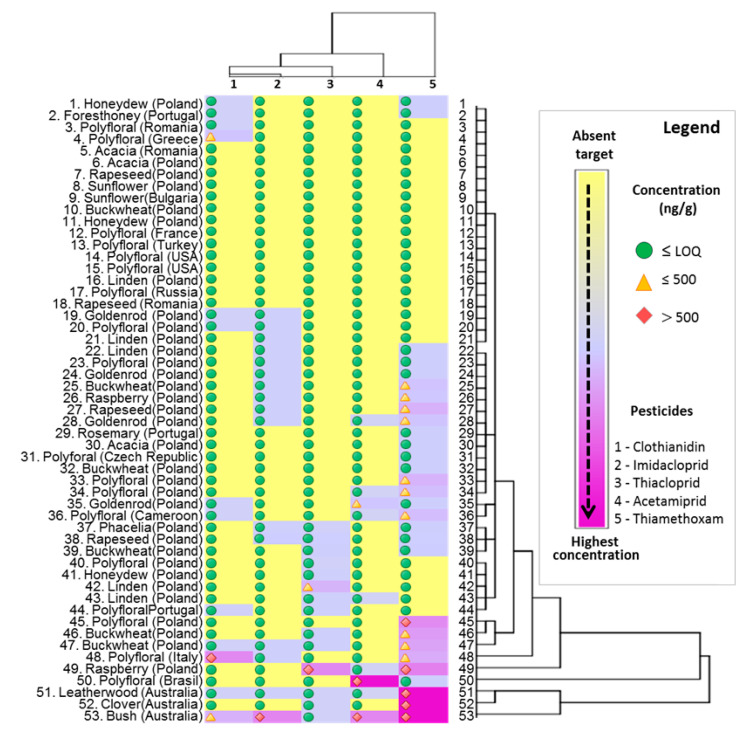
A snapshot of the detected and quantified pesticides in honey samples.

The quantitative results obtained for studied samples of honey are graphically presented in Figure 4, which represent a snapshot of the detected and quantified concentrations of pesticides in honey samples. All the samples in which the investigated neonicotinoids exceeded the LOQ are presented in Figure 4, for a better visualization of detected amounts. Hierarchical clusters analyses presented in Figure 4 (vertical part) classified the samples according with the detected amount of pesticides in various clusters with seven different levels of significance. Moreover, the dendrogram presented in the horizontal part in highlighting that all five investigated pesticides were unevenly distributed in samples. Six of the investigated samples were neonicotinoids free. The sunflower honey was totally free of investigated pesticides. Two samples of rapeseed honey and two samples of acacia honey from Poland and Romania were also neonicotinoids free. In 14 samples the detected pesticides amount was below LOQ (the samples from 5 to 18 presented in Figure 4). Clothianidin and acetamiprid were rarely detected. For samples such as goldenrod, phacelia, some rapeseed, linden, rosemary and buckwheat honey the concentration of the mentioned neonicotinoids did not exceed LOQ value. The most frequently determined compound was thiametoxam and the highest value was determined in the samples from Tasmania, Australia.

In Australia, neonicotinoids are widely used pesticides and this is how their high content in honey samples can be explained. The chemical composition of various types of honey is neither stable nor equal. It depends on many factors such as: the nectar from which honey arises, honey maturity, bee colony that adds enzymes to honey, storage time and conditions and so forth. Different varieties of honey, of even similar types coming from various apiaries may present significant discrepancies in the chemical composition [50]. Nevertheless, regarding the pesticides detected in honey, some reports can be found, which are including neonicotinoids. They are widely applied in the southern part of the world, at high doses [24]. This may explain the high concentration of neonicotinoids in honey originated from Tasmania, Australia. Moreover, in Australia and neighboring countries, neonicotinoids are widely used; but not a high mortality of bee has been observed. Notably, there the bees are less affected by one of a common parasite. There is no Varroa destructor in Australia—a parasite that has long been a nuisance to bees and beekeepers on other continents [18].

**Figure 5:**
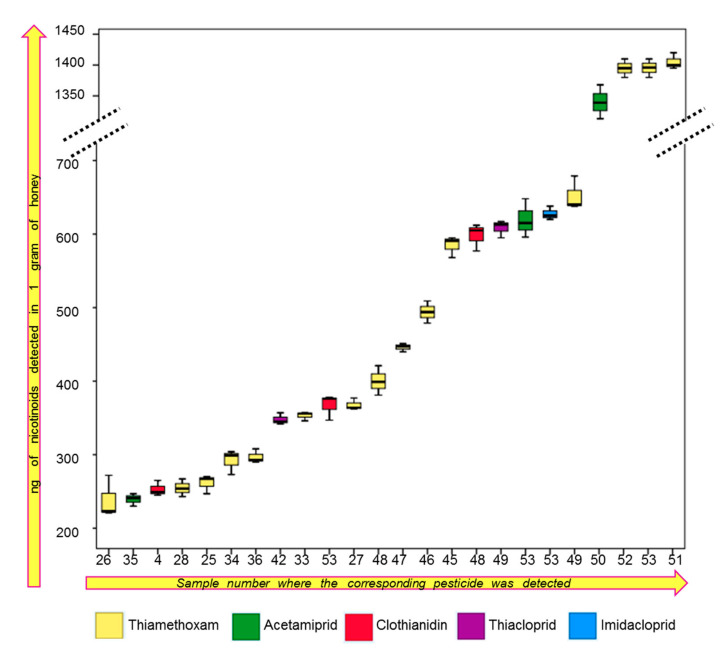
Whiskers box plots presenting the concentration of quantified pesticides which exceeded the LOQ in the investigated samples. The samples number is allotted similar to those presented in Figure 4.

Notwithstanding, the detected amounts of neonicotinoids exceeding the LOQ in all investigated honeys are represented in Figure 5 as whiskers box plots, where the central lines are representing the mean, boxes represent mean ± SD, while whiskers represent min–max values which were calculated for three different replicates. The thiamethoxam detected in all three samples from Australia exceeded 1350 ng/g of honey. Bush honey (sample 53) was the most contaminated. Except for the high concentration of thiamethoxam detected, three more pesticides were measured: clothianidin (367.14 ± 17.29 ng/g), acetamiprid (619.93 ± 26.41) and imidacloprid (624.91 ± 3.16), while the thiacloprid was present below LOQ. High amount of acetamiprid (1340.33 ± 27.50) was detected in the honey originated from Brazil (sample 50). Two neonicotinoids (thiamethoxam and clothianidin) have been also determined in polyfloral honey from Italy (sample 48) with the concentration 400.69 ± 20.29 and 598.84 ± 18.43 respectively. As presented in Figure 4, some samples from Poland were contaminated as well with neonicotinoids, especially thiamethoxam, however the detected concentrations were considerably lower compared with those detailed above. Generally, from 53 investigated samples, the targeted neonicotinoids were present in 19, in higher concentrations than LOQ.

Other methods, like SPE or solvent extraction are also currently used for quantification of neonicotinoids. A comparison of QuEChERS with other two methods, was previously reported in a study related to neonicotinoids quantification in honey and honeybee also [32]. The results highlighted that SPE and solvent extraction were only slightly more sensitive than QuEChERS approach. Nevertheless, QuEChERS approach showed better results than solvent method in the honeybee matrix while SPE was slightly better both in mean recovery and precision than QuEChERS extraction procedure for honey [32]. Considering the limitations of SPE and solvent extraction methods (consumption of materials, sorbents, solvents, long execution time or the large number of steps required to prepare the samples) we can state that QuEChERS present strategical advantages when comparing with the other two methods. 

According with the literature in the field, the pesticides’ presence in honey, including neonicotinoids is clearly stated [24]. QuEChERS approach was also previously optimized and examined for different matrices, including pesticides [52,53,54,55]. Moreover, chemistry for modeling risk assessment on pesticides according with European legislation was also debated in detail in a series of articles written by Villaverde and co-workers [56,57,58,59,60]. In 2013, the use of neonicotinoids in plants attractive to bees was partially banned in Europe Union countries. Actually, EU countries made the decisions on the use of pesticides containing these active substances. Nevertheless, presently the European Commission proposes the implementing by regulations such as 2018/783, 2018/784, 2018/785, where the application of these substances only for use in permanent greenhouses and for the treatment of seeds for the sowing only. Poland, despite the strong efforts of the agricultural lobby, has not issued permission to use them. However, countries such as Finland, Lithuania, Latvia, Estonia, Romania, Hungary and Bulgaria agreed.

## 3. Materials and Methods

### 3.1. Reagents and Standards

Acetamiprid (99.9%) and thiacloprid (99.9%), were purchased from Sigma-Aldrich (St. Louis, MO, USA). Thiamethoxam (99.69%) and imidacloprid (99.5%) were purchased from Witko (Łódź, Poland). Clothianidin (solution in acetonitrile, concentration 100.3 µg/mL) was purchased from Ultra Scientific—Agilent (Santa Clara, CA, USA). All standards of neonicotinoids were stored in a refrigerator. Acetonitrile hypergrade for LC-MS LiChrosolv was obtained from Sigma-Aldrich. Redistilled water was purified with Milli-Q system (Millipore, El Passo, TX, USA). Magnesium sulfate anhydrous, reagent grade ≥99.5% and sodium chloride EMSURE, ACS, ISO Reag. PhEur were purchased from Sigma-Aldrich. Bondesil—primary-secondary amine (PSA) (40 µm, 100 mg) was obtained from Agilent Technologies (Santa Clara, CA, USA). Labstore Baker Bond Octadecyl (C18) Packing 40 µm Prep LC was purchased from J.T.Baker (Deventer, Holland).

### 3.2. Samples Collection

**Figure 6:**
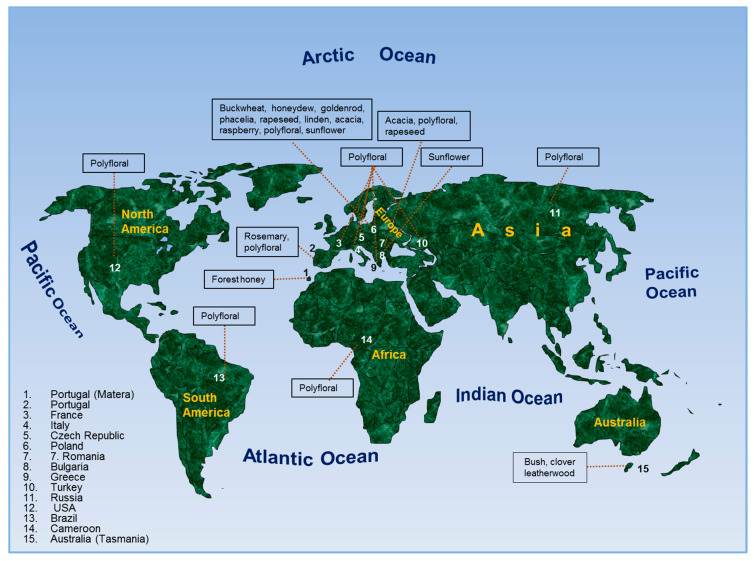
The origin places of honey samples included in the study.

The 53 honey samples of different floral origin (buckwheat, polyfloral, phacelia, rapeseed, linden, sunflower, goldenrod, raspberry, rosemary, bush, leatherwood, clover, acacia, honeydew and forest honey) were collected from different locations in Poland and other countries (Australia, Brazil, Bulgaria, Cameroon, Czech Republic, France, Greece, Italy, Portugal, Romania, Russia, USA, Turkey). All samples were produced in 2019. After collection all samples were stored at room temperature, in the dark. The whole sample collection period took 2 months. The analyses started after all samples were collected. In Figure 6 the origin of collected honey samples has been presented. 

The pollen samples of selected flowers was obtained independently from the beekeepers and were also tested. The conducted investigations included samples such as: buckwheat (*Fagopyrum* Mill.), goldenrod (*Solidago gigantea* L.), phacelia (*Phacelia tanacetifolia* Benth.), rape (*Brassica napus* L. var. *napus*). Pollen samples obtained from selected flowers were characterized with scanning electron microscopy (SEM). The samples were also assessed with energy dispersive X-ray spectroscopy (EDX) to determine the contents of selected elements. The research was developed by Laboratory for Instrumental Analysis (Faculty of Chemistry, NCU, Toruń, Poland) equipped by scanning electron microscopy with EDX spectrometer (SEM-EDX) (SEM, model 1430 VP produced by LEO Electron Microscopy Ltd., Macclesfield, England and EDX, Quantax 200 with XFlash 4010 detector, Bruker AXS, Berlin, Germany). Basic microscope parameters: zoom range from 40 to 800,000 times, theoretical resolution of 5 nm (tungsten cathode), accelerating voltage from 200 V to 30 kV, weight of sample: fractions of gram, sample dimensions: fractions of mm.

### 3.3. Comprehensive Signal Acquisition

The chromatograph (UHPLC 1260 Infinity Agilent Technologies) was equipped with DAD detector. The analytes were separated on a SunShell Phenyl chromatography column (2.1 mm × 150.0 mm × 2.6 μm) at 45 °C. The DAD monitoring wavelengths were set at 245, 254, 265, 270 nm. The mobile phase consisted of water—eluent A and acetonitrile—eluent B. The flow rate was 0.5 mL/min. The injection volume into UHPLC was 5 µL. The mobile phase gradient was programmed as follows: 0 min. 6% B, 5 min. 30% B, 10 min. 41% B, 12 min. 100% B, 15 min. 100% B, 16 min. 6% B, 25 min. 6% B. The time of one analysis was 35 min. 

### 3.4. Sample Preparation

Five grams of honey were weighed and placed in a 50 mL falcon. Ten mL of water was added and mixed on the Vortex for 1 min. 10 mL ACN was added and mixed on the Vortex for 1 min. Then a mixture of the salts 4 g MgSO4 anhydrous and 1 g NaCl was added. The whole was mixed on the Vortex for 1 min and then centrifuged for 5 min on the centrifuge at 3000 rpm. The obtained extracts were analyzed in 2 ways: (1) without purification: 0.5 mL of extract was taken and made the subjected to UHPLC analyses and (2) with purification: 1 mL of extract was taken, purified with a mixture of salts (0.15 g MgSO_4_ anhydrous, 0.05 g PSA and 0.05 g C18 in a 1.5 mL Eppendorf), mixed on Vortex for 30 s, centrifuged for 5 min on the centrifuge at 5000 rpm and then 0.5 mL was taken for UHPLC analysis.

### 3.5. Recovery

Honey was used as blank matrix and spiked by adding the appropriate amount of standard mixture solution of neonicotinoids. Five grams of honey were weighed and placed in a 50 mL falcon. One mL or 0.6 mL of a standard mixture of acetonitrile at 2 µg/mL for acetamiprid, imidacloprid, clothianidin, thiamethoxam and 5 µg/mL for thiacloprid was added. They were mixed on the Vortex and put in the fridge overnight. Ten mL of water was added and mixed on the Vortex for 1 min. Nine mL or 9.4 mL ACN was added and mixed on the Vortex for 1 min. Then a mixture of the salts of ECMSSC-MP (Mylar pouch containing 4 g MgSO_4_ anhydrous and 1 g NaCl) was added. The whole content was mixed on the Vortex for 1 min and then centrifuged for 5 min at 3000 rpm. The obtained extracts were analyzed in 2 ways: (1) without purification: 0.5 mL of extract was taken and analyzed by UHPLC; and (2) with purification as described in sample preparation section.

### 3.6. Method Validation

Individual standard stock solutions of 10.0 mg/mL were prepared by dissolving 100.0 mg of the respective analyte (with the exception of clothianidin) in the appropriate amount of acetonitrile (volumetric flask, 10 mL). The standard solutions were diluted by transferring 100 µL of acetamiprid, thiamethoxam, imidacloprid and 250 µL of thiacloprid into a 5 mL flask. The standard mix solution at 2.0 µg/mL of standard pesticides (acetamiprid, thiamethoxam, imidacloprid) and 5.0 µg/mL of thiacloprid was diluted by transferring 100 µL of mix standard and 200 µL of clothianidin into a volumetric flask (10 mL). The standard stock solutions were diluted to obtain mixture working solutions of all investigated analytes. All mix solutions were making up at volume with acetonitrile. The calibrations curves for five neonicotinoids were performed ranging from 100 to 700 ng/mL for all analytes except for thiacloprid. For thiacloprid the calibration curve was from 150 to 1700 ng/mL. The standards solutions were protected from light and stored in a freezer (−20 °C).

The calibrations curves were constructed for each standard at the appropriate wavelength: λ = 245 nm for acetamiprid and thiacloprid, λ = 254 nm for thiamethoxam, λ = 265 nm for clothianidin, λ = 270 nm for imidacloprid. The LOD and LOQ values were estimated based on the standard deviation (SD) of the response of the curve and the slope of the calibration curve (S). The LOD was calculated according to equation: LOD = 3.3 · SD/S and LOQ = 10 · SD/S. SD of the response was designated based on the SD of y-intercepts of regression lines.

### 3.7. Determination of Physicochemical Properties

#### 3.7.1. Pfund Value

Four grams of honey was dissolved 8 mL of warm distilled water (50 °C). The absorbance of the obtained mixture was measured at wave length 635 using a UV-Vis spectrophotometer (NanoDrop 2000c; Thermo Fisher Scientific, Waltham, MA, USA). Each sample was measured in triplicate. The Pfund value was calculated with the Equation (2).

Pfund [mm] = −38.7 + 371.39 × Abs(2)

where: Pfund = honey color according with Pfund scale; Abs = absorbance at λ = 635 nm.

#### 3.7.2. Acidity and pH

Five grams of each honey type was dissolved in 37.5 mL of distilled water. The pH of the obtained solutions was measured by a pH-meter (CPC-501; Elmetron, Chorzow, Poland) with a glass electrode. Each sample was measured in triplicate.

The acidity was determined by involving a potentiometric titration of the solutions prepared for pH measurement. Each honey solution was titrated with NaOH (0.1 M) until the pH value of 8.3 was obtained. The acidity was calculated based on the Equation (3). The obtained values were expressed in milliequivalent of acid per kilogram of honey.

A [meq/kg] = VNaOH × 10(3)

where: A = total acidity; VNaOH = the volume of NaOH (0.1 M) used for the titration.

#### 3.7.3. Electrical Conductivity

Two grams of honey was dissolved in 10 mL of deionized water. Aliquots of 1 mL was taken from each solution and cooled down to 20 °C. After the temperature was adjusted, the solution was transferred in the conductivity cell and the electrical conductivity was measured (with the device CPC-501 (Elmetron, Chorzów, Poland)). Each sample was measured in triplicate.

### 3.8. Statistical Analyses

The world map was drawn in PowerPoint 2010. Hierarchical clustering analysis and, correlation analysis and the box plots were created using IBM SPSS Statistical package, version 21. Microsoft Excel 2016 and Microsoft Power Point 2010 were used to for routine calculation, as well as in preparation and integration of the figures composed from multiple parts

## 4. Conclusions

The method consisting of QuEChERS as a sample preparation and analysis of extracts by means of UHPLC/UV gave reproducible results and high recoveries. In this paper an analytical method for the separation and determination of five neonicotinoids in various origin honey samples have been reported. The proposed method is relatively fast and easy to perform. Many of analyzed samples were characterized by a lack of thiamethoxam, clothianidin, imidacloprid, acetamiprid and thiacloprid or concentrations of these compounds were at the LOD and LOQ levels. High concentrations and the presence of these compounds were observed in honey from Tasmania. The proposed analytical method could be a useful tool for the routine monitoring of honeys for residues of neonicotinoid insecticides due to the use of QuEChERS method as well as UHPLC/UV—cheaper compared to classic HPLC coupled with mass spectrometry technique.

Future work related to QuEChERS method for pesticides quantification in honey will be directed to a deep optimization of both sample preparation method and chromatographic analysis. The mentioned optimization will be realized using response surface methodology with Box-Behnken design, as we previously developed for investigating extraction parameters in accelerated solvent extraction [61] and pressurized liquid extraction [62] that were designed for isolation of sugars and cyclitols from plant material.

## Figures and Tables

**Table 1:** General characteristics of pollen grains of selected plants.

Name (*Latin Name*) and Picture	SEM	Elements & EDX Results [%]	
Buckwheat (*Fagopyrum Mill.*) 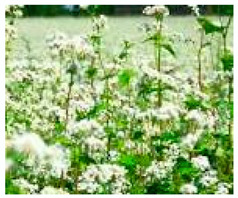	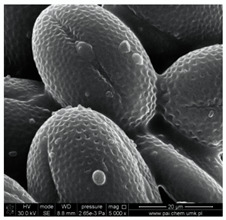	C	26.54
		O	68.53
		Na	0.45
		Mg	0.33
		Si	0.21
		P	0.72
		S	0.21
		Cl	0.01
		K	2.08
		Ca	0.98
Goldenrod (*Solidagogigantea L.*) 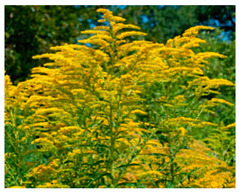	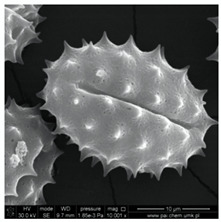	C	29.86
		O	68.03
		Na	0.02
		Mg	0.00
		Si	0.00
		P	0.34
		S	0.16
		Cl	0.14
		K	0.67
		Ca	0.79
Phacelia (*Phacelia tanacetifolia* Benth.) 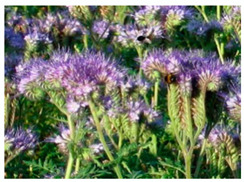	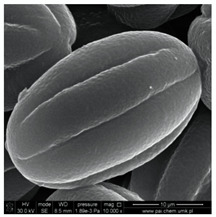	C	21.44
		O	69.06
		Na	0.40
		Mg	0.59
		Si	0.69
		P	0.31
		S	0.55
		Cl	0.26
		K	5.21
		Ca	1.41
Rape (*Brassica napus* L. var. *napus*) 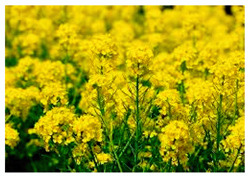	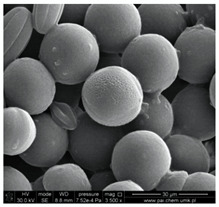	C	25.13
		O	70.91
		Na	0.69
		Mg	0.64
		Si	-
		P	0.47
		S	0.20
		Cl	0.16
		K	1.56
		Ca	0.23

**Table 2:** Results of method validation, where: 1 = thiamethoxam, 2 = clothianidin, 3 = imidacloprid, 4 = acetamiprid, 5 = thiacloprid.

Target	Structural Formula	Linearity [ng/mL]	Calibration Curve		R^2^	LOD [ng/g]	LOQ [ng/g]	Recovery ± SD [%] (First Level: 120 & 300 ng/mL)	Recovery ± SD [%] (Second Level: 200 & 500 ng/mL)
			a	b					
1	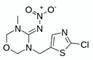	99.69–697.83	0.0346	−0.0388	0.9995	65.2	197.6	86.17 ± 8.77	99.64 ± 5.61
2	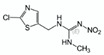	100.30–702.10	0.0442	−0.4734	0.9994	63.1	191.1	101.71 ± 3.10	101.06 ± 3.04
3	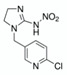	99.50–696.50	0.0474	−0.0264	0.9993	64.7	195.9	98.34 ± 2.21	95.30 ± 5.82
4	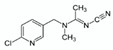	99.90–699.30	0.0533	0.4870	0.9996	60.8	184.3	100.62 ± 1.34	95.20 ± 5.56
5	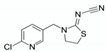	149.85–1748.25	0.0437	−0.0067	0.9998	81.0	245.4	101.63 ± 0.90	101.60 ± 3.54

**Table 3:** Thiamethoxam, clothianidin, imidacloprid, acetamiprid and thiacloprid contents of honeys of different botanical origin (*n* = 3); where: nd—not detected.

No	Honey	The Neonicotinoids Content in Honey [ng/g]				
		Thiamethoxam	Clothianidin	Imidacloprid	Acetamiprid	Thiacloprid
1	Goldenrod (Poland)	Nd	<LOQ	<LOQ	nd	nd
2	Goldenrod (Poland)	<LOQ	nd	<LOQ	nd	nd
3	Goldenrod (Poland)	254.97 ± 11.96	nd	<LOQ	<LOQ	nd
4	Goldenrod (Poland)	<LOQ	<LOQ	nd	242.82 ± 3.66	nd
5	Phacelia (Poland)	<LOQ	nd	<LOQ	nd	<LOQ
6	Rape (Poland)	368.26 ± 8.36	nd	<LOQ	nd	nd
7	Rape (Poland)	<LOQ	nd	<LOQ	nd	<LOQ
8	Rape (Poland)	Nd	nd	nd	nd	nd
9	Rape (Romania)	Nd	nd	nd	nd	nd
10	Linden (Poland)	<LOQ	nd	<LOQ	nd	nd
11	Linden (Poland)	Nd	nd	nd	nd	348.19 ± 8.30
12	Linden (Poland)	Nd	nd	<LOQ	nd	nd
13	Linden (Poland)	Nd	nd	nd	<LOQ	<LOQ
14	Linden (Poland)	Nd	nd	nd	nd	nd
15	Multifloral (Poland)	292.38 ± 16.67	nd	nd	<LOQ	nd
16	Multifloral (Poland)	<LOQ	nd	<LOQ	nd	nd
17	Multifloral (Poland)	353.17 ± 5.97	nd	nd	nd	nd
18	Multifloral (Poland)	591.28 ± 6.14	nd	nd	nd	nd
19	Multifloral (Poland)	Nd	<LOQ	<LOQ	nd	nd
20	Multifloral (Poland)	Nd	nd	nd	nd	<LOQ
21	Multifloral (Romania)	Nd	<LOQ	nd	nd	nd
22	Multifloral (Czech Republic)	<LOQ	nd	nd	nd	nd
23	Multifloral (Russia)	Nd	nd	nd	nd	nd
24	Multifloral (Greece)	Nd	253.39 ± 10.49	nd	nd	nd
25	Multifloral (Brasil)	<LOQ	nd	nd	1340.33 ± 27.50	nd
26	Multifloral (USA)	Nd	nd	nd	Nd	nd
27	Multifloral (USA)	Nd	nd	nd	Nd	nd
28	Multifloral (Cameroon)	297.39 ± 9.69	<LOQ	nd	<LOQ	nd
29	Multifloral (France)	Nd	nd	nd	Nd	nd
30	Multifloral (Portugal)	Nd	<LOQ	nd	nd	<LOQ
31	Multifloral (Italy)	400.69 ± 20.29	598.84 ± 18.43	<LOQ	nd	nd
32	Multifloral (Turkey)	Nd	nd	nd	nd	nd
33	Buckwheat (Poland)	Nd	nd	nd	nd	nd
34	Buckwheat (Poland)	494.47 ± 14.83	nd	nd	nd	<LOQ
35	Buckwheat (Poland)	261.85 ± 12.60	nd	<LOQ	nd	nd
36	Buckwheat (Poland)	<LOQ	nd	nd	nd	<LOQ
37	Buckwheat (Poland)	447.81 ± 4.52	<LOQ	<LOQ	nd	<LOQ
38	Buckwheat (Poland)	<LOQ	nd	nd	nd	nd
39	Honeydew (Poland)	Nd	nd	nd	nd	<LOQ
40	Honeydew (Poland)	Nd	nd	nd	nd	nd
41	Honeydew (Poland)	<LOQ	<LOQ	nd	nd	nd
42	Raspberry (Poland)	239.46 ± 28.96	nd	<LOQ	nd	nd
43	Raspberry (Poland)	652.42 ± 23.06	nd	nd	<LOQ	608.40 ± 11.60
44	Sunflower (Poland)	Nd	nd	nd	Nd	nd
45	Sunflower (Bulgaria)	Nd	nd	nd	Nd	nd
46	Rosemary (Portugal)	<LOQ	nd	nd	Nd	nd
47	Bush (Australia, Tasmania)	presumably ≥ 1395.66	367.14 ± 17.29	624.91 ± 3.16	619.93 ± 26.41	<LOQ
48	Acacia (Romania)	Nd	nd	nd	Nd	nd
49	Acacia (Poland)	Nd	nd	nd	nd	nd
50	Acacia (Poland)	<LOQ	nd	nd	nd	nd
51	Leatherwood (Australia, Tasmania)	presumably ≥ 1395.66	<LOQ	<LOQ	<LOQ	<LOQ
52	Clover (Australia, Tasmania)	presumably ≥ 1395.66	nd	nd	nd	<LOQ
53	Foresthoney (Portugal, Madera)	<LOQ	<LOQ	nd	nd	nd
